# Long Non-Coding RNAs in HER2-Positive Breast Cancer: From Resistance Mechanisms to Translational Potential

**DOI:** 10.32604/or.2026.075346

**Published:** 2026-05-21

**Authors:** Thanh Hoa Vo, Edel McNeela, Orla O’Donnovan, Jai Prakash Mehta, Van Hoa Nguyen, Sweta Rani

**Affiliations:** 1 Department of Science, South East Technological University, Waterford, Ireland; 2 Pharmaceutical and Molecular Biotechnology Research Centre (PMBRC), Waterford, Ireland; 3 Department of Applied Science, South East Technological University, Carlow, Ireland; 4 Department of Radiotherapy, Da Nang Oncology Hospital, Da Nang, Vietnam

**Keywords:** LncRNAs, human epidermal growth factor receptor 2 (HER2)-positive breast cancer, drug resistance, exosomes, liquid biopsy, CRISPR–Cas13, biomarkers

## Abstract

Long non-coding RNAs (lncRNAs) have emerged as key regulators of drug resistance in human epidermal growth factor receptor 2 (HER2)-positive breast cancer, a subtype in which both intrinsic and acquired resistance to HER2-targeted therapies remain major clinical challenges. Although mechanistic studies have begun to reveal how lncRNAs modulate signaling pathways, interact with microRNAs, and influence the tumor microenvironment, dedicated investigations in HER2-positive disease are still limited. This review synthesizes current evidence across epigenetic, transcriptional, and post-transcriptional mechanisms of resistance, including competing endogenous RNA (ceRNA) networks, RNA-binding protein interactions, and exosome-mediated intercellular communication. Particular emphasis is given to resistance-associated lncRNAs such as HOX transcript antisense RNA (HOTAIR), long intergenic non-protein coding RNA 969 (LINC00969), and growth arrest-specific 5 (GAS5), which exemplify the diverse molecular strategies underlying therapy evasion. We further discuss the emerging translational potential of lncRNAs as liquid-biopsy biomarkers, therapeutic targets for antisense oligonucleotides or CRISPR-Cas13 platforms, and cargo for HER2-targeted exosome delivery. Integrating exosomal lncRNA profiling with circulating tumor DNA (ctDNA) monitoring could enable earlier detection of resistance and inform adaptive treatment strategies. By combining mechanistic insight with translational outlook, this review positions lncRNAs as promising yet underexplored contributors to HER2-positive breast cancer drug resistance and outlines a roadmap for advancing their clinical utility. The aim of this review is to synthesize current evidence on lncRNA-mediated resistance mechanisms in HER2-positive breast cancer and to highlight translational opportunities for lncRNA-based biomarkers and therapeutic strategies.

## 1 Introduction

Human epidermal growth factor receptor 2 (HER2)-positive breast cancer accounts for approximately 15–20% of cases and is characterized by distinct molecular features and more aggressive clinical behavior compared with other subtypes [1,2,3]. The development of HER2-directed agents, including trastuzumab [4], pertuzumab [5], tyrosine kinase inhibitors [6,7,8], and antibody-drug conjugates [9,10], has significantly improved patient outcomes. Nonetheless, both intrinsic and acquired resistance remain major barriers, limiting the durability of clinical benefit [11,12,13]. Beyond canonical resistance pathways, long non-coding RNAs (lncRNAs) have emerged as critical regulators with implications for both disease biology and potential therapeutic intervention.

LncRNAs are endogenous RNA transcripts longer than 200 nucleotides that lack significant open reading frames, which is why they lack substantial protein-coding potential [14,15]. Due to their diverse origins and functions, lncRNAs represent a highly heterogeneous class of RNA molecules. Most lncRNAs are transcribed by RNA polymerase II and occasionally by other polymerases [16]. Following transcription, lncRNAs typically undergo canonical RNA-processing steps, including 5′ capping, splicing, and polyadenylation. Compared to protein-coding transcripts, lncRNAs typically exhibit lower expression. Despite their low abundance, many lncRNAs possess conserved promoter regions, intronic or exonic sequences, and stable secondary structures that contribute to their regulatory functions [17].

Dysregulation of lncRNAs has been implicated in a wide range of diseases, particularly cancer [18]. They play critical roles in regulating transcriptional and post-transcriptional processes of protein-coding genes. Additionally, lncRNAs influence chromatin architecture and mediate interactions among proteins, DNA, and other RNAs [19,20]. Emerging studies have revealed that lncRNAs participate in various cellular processes through distinct mechanisms, including acting as molecular signals, decoys, guides, and scaffolds [21,22]. Furthermore, some lncRNAs serve as precursors for microRNAs (miRNAs), which are themselves key regulators of tumorigenesis [23,24].

Recent studies have increasingly investigated the pivotal role of lncRNAs in breast cancer, linking their dysregulation to tumorigenesis, diagnostic potential, and therapeutic resistance across molecular subtypes [25,26,27]. LncRNAs have been shown to influence drug response by modulating key signaling pathways, interacting with microRNAs, and reshaping the tumor microenvironment (TME) [28,29,30]. In HER2-positive breast cancer, several lncRNAs have been associated with drug resistance. HOX Transcript Antisense Intergenic RNA (HOTAIR) promotes epigenetic silencing of phosphatase and tensin homolog (PTEN) via Polycomb Repressive Complex 2 (PRC2) recruitment, resulting in PI3K/AKT activation and epithelial–mesenchymal transition (EMT) [31,32]. LINC00969 enhances the stability of HER2 mRNA through interaction with the RNA-binding protein human antigen R (HuR) [33], while growth arrest-specific 5 (GAS5) restores PTEN expression by sponging miR-21, thereby counteracting resistance [34,35]. In addition, exosomal lncRNAs such as LINC00969 have been detected in plasma, where their expression correlates with therapy resistance and clinical outcome, highlighting their potential as liquid biopsy biomarkers [33,36]. Additionally, HER2-positive breast cancer is clinically heterogeneous, and hormone receptor (HR) status (HR+/HER2+ vs. HR−/HER2+) can shape signaling dependencies and the tumor microenvironment. Accordingly, the clinical performance and prioritization of lncRNA biomarkers and targets may differ across HER2-positive subgroups and warrants stratified evaluation in future validation studies.

This review synthesizes current evidence on how lncRNAs contribute to resistance in HER2-positive breast cancer, focusing on their roles in epigenetic regulation, transcriptional and post-transcriptional modulation, and intercellular communication within the TME through exosomes. We also examine translational opportunities, including the potential of lncRNAs as circulating biomarkers, as therapeutic targets for antisense oligonucleotides or CRISPR-Cas13-based approaches, and as cargo for HER2-targeted exosome delivery systems. Finally, we discuss how combining exosomal lncRNA analysis with ctDNA monitoring may enhance early detection of resistance and provide a framework for adaptive treatment strategies.

The aim of this review is to summarize current evidence on lncRNA-mediated mechanisms of resistance to HER2-targeted therapies in HER2-positive breast cancer and to highlight translational opportunities for lncRNA-based biomarkers and therapeutic strategies.

## 2 Mechanisms of LncRNAs in Mediating HER2-Targeted Drug Resistance

In HER2-positive breast cancer, lncRNAs mediate drug resistance through multiple mechanisms. These include epigenetic and transcriptional regulation of HER2 (ERBB2; erb-b2 receptor tyrosine kinase 2) and associated pathways, post-transcriptional control via miRNA sponging or mRNA stability modulation, and reshaping the TME, including immune modulation and intercellular signaling through exosomes [37,38]. These functions operate at transcriptional, post-transcriptional, and epigenetic levels, influencing processes such as EMT, autophagy, immune evasion, and cellular stress responses [36,39]. Understanding these mechanisms is essential for overcoming resistance to HER2-targeted therapies.

### 2.1 Epigenetic and Transcriptional Regulation

LncRNAs contribute to drug resistance in HER2-positive breast cancer by regulating the transcription of oncogenes, tumor suppressors, and critical signaling pathway components [37,40]. They can modulate transcription factor activity and chromatin accessibility, thereby altering the expression of genes that influence therapeutic response [22,32,38]. Such regulation may enhance HER2-driven oncogenic signaling or suppress genes that mediate sensitivity to HER2-targeted therapies.

HOTAIR is one of the most studied lncRNAs in HER2-positive breast cancer drug resistance. HOTAIR is a well-characterized lncRNA transcribed from the HOXC locus and has been widely implicated in cancer progression and therapy resistance [41,42,43,44]. The HOTAIR promoter contains binding sites for activator protein 1 (AP-1) and specificity protein 1 (Sp1)-transcription factors (TFs) that regulate promoter activity and gene expression. It also harbors estrogen-responsive and hypoxia-responsive elements, allowing HOTAIR expression to be induced by hormonal and stress signals commonly observed in tumors [41,42].

HOTAIR acts as a scaffold for the PRC2, which includes the histone methyltransferase zeste homolog 2 (EZH2), and for the REST corepressor (CoREST) complex containing lysine-specific demethylase 1 (LSD1). Through PRC2 recruitment, HOTAIR promotes trimethylation of histone H3 at lysine 27 (H3K27me3) at tumor suppressor loci [43,44]. In trastuzumab-resistant HER2-positive breast cancer cells, HOTAIR has been reported to contribute to PTEN silencing, a key negative regulator of the PI3K/Akt pathway, thereby promoting drug resistance [31,41,42,43,44]. However, the requirement for PRC2 in HOTAIR-mediated transcriptional repression appears to be context-dependent, and PRC2-independent effects have also been reported [32]. This epigenetic silencing indirectly enhances HER2 signaling by removing negative regulators of proliferation and survival. In HER2-positive breast cancer cells, HOTAIR is frequently upregulated and correlates with poor prognosis and resistance to trastuzumab [41].

In addition to chromatin remodeling, HOTAIR has been associated with EMT marker changes, including increased TGF-β, vimentin, and Snail expression and reduced E-cadherin levels, and proliferation in resistant HER2-positive breast cancer cells, further supporting its role as a central regulator of therapy resistance [41].

### 2.2 Post-Transcriptional Modulation

In HER2-positive breast cancer, several lncRNAs modulate post-transcriptional control by acting as competing endogenous RNAs (ceRNAs) that sequester miRNAs targeting HER2-related transcripts or downstream signaling genes. In HER2-positive breast cancer, lncRNA GAS5 has been reported to sponge miR-21, thereby restoring PTEN expression and inhibiting PI3K/Akt signaling, which can influence trastuzumab sensitivity [34,35]. These ceRNA interactions can modulate HER2-driven oncogenic signaling and influence the response to HER2-targeted therapies, either promoting resistance or enhancing sensitivity depending on the specific lncRNA–miRNA–mRNA axis involved.

Certain lncRNAs influence the stability of HER2-related mRNAs by interacting directly with the transcripts or through RNA-binding proteins (RBPs). For example, LINC00969 can bind to the RBP HuR (ELAVL1), enhancing the stability of HER2 mRNA and promoting sustained HER2 expression [33]. Other lncRNAs may recruit RBPs that facilitate the degradation of tumor-suppressive transcripts, contributing to a pro-oncogenic environment [45]. By preserving HER2 signaling and associated survival pathways, lncRNA–RBP interactions may contribute to reduced responsiveness to HER2-targeted therapies such as trastuzumab [46].

### 2.3 Tumor Microenvironment (TME) Influence

Several lncRNAs can shape the immune landscape in breast cancer. For instance, SNHG1 and LINC00514 promote M2-like macrophage polarization, which suppresses anti-tumor immunity [47,48]. In HER2-positive disease, however, direct links between these lncRNA-driven macrophage states and reduced efficacy of trastuzumab/other HER2-directed antibodies (including Fc-mediated ADCC/ADCP) remain to be established. Key next steps include trastuzumab-based co-culture assays measuring ADCC/ADCP with natural killer (NK) cells and macrophages after SNHG1 or LINC00514 perturbation, followed by confirmation in immune-competent or humanized *in vivo* models. Accordingly, immune polarization is presented here as an emerging modulatory layer of response to Fc-enabled anti-HER2 antibodies, whereas canonical tumor-intrinsic mechanisms such as PI3K/AKT pathway activation and HER2 heterogeneity remain more established drivers of clinical resistance.

LncRNAs are actively packaged into tumor-derived exosomes, facilitating intercellular communication. Exosome-associated RNAs have been explored as circulating biomarkers in breast cancer, and exosome-based liquid biopsy approaches have been proposed for early detection and disease monitoring [30,49]. Moreover, elevated exosomal HOTAIR has been reported to be associated with poor survival and diminished chemotherapy response [36,50,51]. Despite limited direct evidence in HER2-targeted therapy resistance, emerging data suggest that exosomal lncRNAs, such as HOTAIR, may serve as both biomarkers and functional mediators of resistance. A summary of key lncRNAs implicated in HER2-positive breast cancer resistance, their mechanisms, and supporting evidence is provided in Table 1.

**Table 1:** Key lncRNAs implicated in HER2-positive breast cancer resistance, categorized by mechanism of action.

Mechanism	LncRNA	Mode of Action	Molecular Target/Pathway	Experimental Evidence	Reference
Epigenetic and transcriptional regulation	HOTAIR	Recruits PRC2/EZH2, silences PTEN; promotes EMT	PI3K/AKT, EMT	Trastuzumab-resistant HER2+ cells; clinical correlation	[31,32,41,42,43,44]
Post-transcriptional modulation (ceRNA)	GAS5	Sponges miR-21 → restores PTEN	PI3K/AKT	*In vitro* models; trastuzumab sensitivity	[34,35]
Post-transcriptional modulation (RBP)	LINC00969	Binds HuR (ELAVL1), stabilizes HER2 mRNA → sustained HER2 expression	ERBB2/HER2 signaling	Trastuzumab-resistant HER2+ cell models	[33]
TME–immune	SNHG1	Promotes M2-like macrophage polarization	Immune evasion	Functional evidence in breast cancer	[47,48]
TME–immune	LINC00514	Promotes M2-like macrophage polarization	Immune evasion	Functional evidence in breast cancer	[47,48]
TME–exosomal	HOTAIR	Packaged into exosomes; transferred to recipient cells, potentially contributing to EMT-associated and resistance-linked programs	PTEN/PI3K/AKT, EMT	Detected in serum/plasma; reported clinical association	[30,36,49,50,51]
TME–exosomal	LINC00969	Packaged into exosomes; delivered to recipient cells, where it binds HuR and stabilizes HER2 mRNA	ERBB2/HER2 stabilization	Detected in plasma of HER2+ patients; correlates with trastuzumab resistance	[30,33,49]

Abb: Polycomb repressive complex 2 (PRC2); enhancer of zeste homolog 2 (EZH2); phosphatase and tensin homolog (PTEN); epithelial–mesenchymal transition (EMT); phosphoinositide 3-kinase (PI3K); protein kinase B (AKT); competing endogenous RNA (ceRNA); RNA-binding protein (RBP); human antigen R (HuR; ELAVL1); human epidermal growth factor receptor 2 (HER2); tumor microenvironment (TME); microRNA (miRNA); erb-b2 receptor tyrosine kinase 2 (ERBB2).

## 3 LncRNAs as Nodes in Multi-Layer Regulatory Networks of HER2 Drug Resistance

Biological regulation often occurs through networks, interconnected systems of biomolecules such as genes, RNAs, and proteins, where the edges define how one component influences another, and the nodes may interact with multiple partners. Unlike linear pathways, networks capture the complexity, redundancy, and adaptability of cellular regulation, where connectivity between nodes allows multiple layers of influence, feedback, and feed-forward loops to fine-tune regulatory signals, and robustness ensures that biological functions are maintained through alternative routes if one node is disrupted [52]. In the context of HER2-positive breast cancer, such network properties may enable malignant cells to bypass the inhibition of a single pathway, such as HER2 blockade, by sustaining oncogenic signaling through alternative routes or compensatory mechanisms. While comprehensive network-level analyses of HER2-resistant models are still emerging, preliminary data highlight the involvement of multiple lncRNAs and miRNAs converging on key pathways like PI3K/Akt and EMT, suggesting that a network perspective could illuminate novel combinatorial therapeutic strategies [53,54].

### 3.1 LncRNA-miRNA-mRNA Interactomes (ceRNA Networks)

At a systems level, lncRNAs can participate in complex competing endogenous RNA (ceRNA) networks, where they sponge multiple miRNAs to influence mRNA expression and cellular behavior. This ceRNA crosstalk forms multilayered networks. A broad ceRNA network can be constructed to map lncRNA-miRNA-mRNA interactions related to drug resistance across cancers. Liu et al. identified 83 lncRNAs and 379 mRNAs involved in drug resistance modules across multiple cancer types and therapeutics, revealing shared resistance mechanisms and suggesting ceRNA modules as novel biomarkers [53]. Disruption of ceRNA networks, rather than single miRNA or lncRNA, can drive drug resistance [55,56].

In HER2-positive breast cancer, several experimentally validated lncRNA-miRNA-mRNA axes exemplify how ceRNA networks operate. Rather than functioning through isolated interactions, these networks integrate multiple lncRNAs and miRNAs that collectively regulate key signaling pathways, including PI3K/AKT and EMT programs. Such ceRNA crosstalk allows cancer cells to dynamically fine-tune survival and resistance signaling, providing regulatory redundancy that buffers against HER2 inhibition. Viewing HER2-targeted resistance through this network-based framework highlights the complex interdependencies among non-coding RNAs and offers a systems-level understanding of how therapeutic responses are maintained or circumvented under drug pressure.

### 3.2 LncRNAs as Integrated Regulators of Chromatin and Post-Transcriptional Control

Some lncRNAs, exemplified by HOTAIR, display dual functionality. As reviewed in the previous section, HOTAIR engages in chromatin remodeling via PRC2/EZH2, leading to the repression of tumor suppressors such as PTEN. Other studies in different cancer contexts have shown that lncRNAs can also influence miRNA activity through methylation-mediated silencing of miRNA genes [57]. These combined activities position such lncRNAs as critical network hubs that bridge epigenetic and post-transcriptional regulatory layers, making their modulation particularly impactful in the context of therapy resistance.

### 3.3 Network Convergence on Key Oncogenic Pathways

In HER2-positive breast cancer, multiple lncRNA–miRNA modules converge on canonical downstream signaling axes, particularly the PI3K/Akt and EMT pathways. For PI3K/Akt, there is evidence of lncRNAs exerting opposing effects on the same pathway: GAS5 sponging of miR-21 restores PTEN expression, weakens PI3K/Akt signaling, and enhances trastuzumab sensitivity [34,35], whereas HOTAIR epigenetically silences PTEN via PRC2/EZH2 recruitment, activating PI3K/Akt/mammalian target of rapamycin (mTOR) signaling and promoting resistance [31,41]. In contrast, EMT-associated lncRNA mechanisms in HER2-positive resistance remain less well mapped and require additional validation.

### 3.4 Intercellular Networks via Exosomal LncRNAs

**Figure 1:**
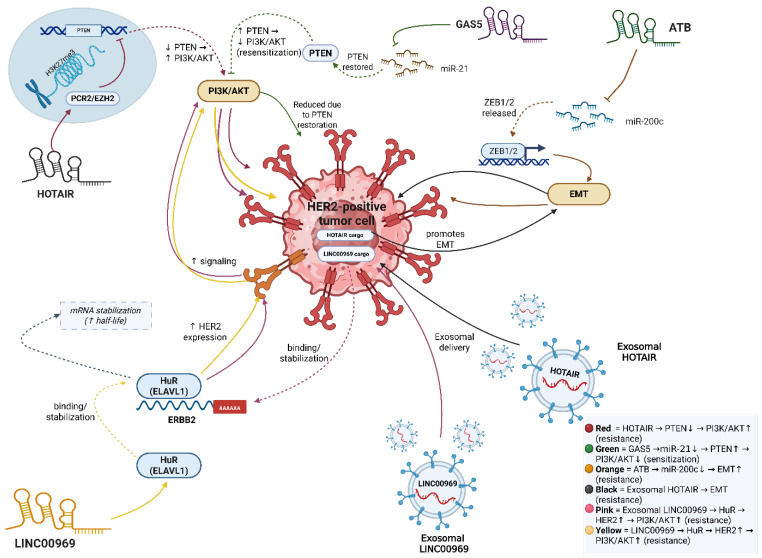
LncRNA-mediated regulatory networks in HER2-positive breast cancer drug resistance. Schematic representation of lncRNAs modulating HER2 resistance through ceRNA crosstalk, chromatin remodeling, and exosomal transfer, converging on PI3K/AKT and EMT pathways. This figure was created using BioRender. Abb: human epidermal growth factor receptor 2 (HER2), phosphoinositide 3-kinase/protein kinase B (PI3K/AKT), epithelial–mesenchymal transition (EMT).

Regulatory networks in HER2-positive breast cancer are not confined to intracellular interactions; they also operate between cells via extracellular vesicles (EVs) such as exosomes. Exosomal lncRNAs function as intercellular messengers, transferring regulatory signals from drug-resistant to drug-sensitive cells. This transfer can reprogram gene expression in recipient cells, for instance, by increasing HER2 expression or inducing EMT, thereby facilitating the emergence of resistant phenotypes in previously sensitive populations. In HER2-positive breast cancer, exosomal LINC00969 binds the RNA-binding protein HuR (ELAVL1), stabilizing ERBB2 mRNA and sustaining HER2 overexpression, ultimately conferring trastuzumab resistance [33]. Circulating exosomal HOTAIR is elevated in the circulation of breast cancer patients, correlates with HER2 positivity, and associates with poorer survival/chemotherapy response [50,51]. While direct demonstrations that HOTAIR-containing exosomes induce EMT or angiogenesis in breast cancer are still limited, exosomes in general have been shown to promote EMT and other resistance-linked programs in breast cancer, underscoring an intercellular layer of regulation relevant to therapy response [33,36]. Through such mechanisms, exosomal lncRNAs extend the regulatory network into the TME, with cancer cells acting as nodes and exosomal communication forming the edges of an intercellular resistance network. Fig. 1 illustrates the lncRNA-mediated regulatory networks in HER2-positive breast cancer drug resistance.

## 4 Translational Potential of LncRNAs in HER2-Positive Breast Cancer Drug Resistance

The integration of lncRNA research into clinical decision-making for HER2-positive breast cancer remains at an early stage, but emerging evidence highlights several candidates with potential biomarker and therapeutic utility. This section explores some translational avenues, including using lncRNAs as clinical biomarkers for baseline risk stratification and on-treatment monitoring and therapeutic targeting of resistance-associated lncRNAs to restore response to HER2-directed therapy.

### 4.1 Clinical Biomarker Potential

Although evidence largely stems from preliminary studies, a few lncRNAs implicated in resistance mechanisms demonstrate promise as biomarkers of therapy response in clinical settings. Circulating exosomal LINC00969 has emerged as a potential liquid biopsy marker in HER2-positive breast cancer, its elevated plasma levels correlate with trastuzumab resistance, and monitoring its changes during therapy may be explored as an early indicator of treatment failure in future prospective studies [33]. Similarly, exosomal HOTAIR was reported as elevated at diagnosis in HER2-positive breast cancer and is associated with poorer survival and diminished chemotherapy responsiveness [36]. In a clinical context, baseline HOTAIR measurement could be explored for risk stratification in future prospective studies to identify patients who may require closer monitoring or combination regimens from the outset. Furthermore, dynamic changes in circulating HOTAIR levels during treatment might serve as an early pharmacodynamic readout, contributing to adaptive therapy strategies.

Beyond these examples, broader transcriptomic profiling of circulating lncRNAs could help identify patient-specific resistance signatures. Such panels, once validated in large, prospective trials, may provide multi-lncRNA risk scores, improving predictive accuracy compared with single-marker assays. This approach is supported in other breast cancer contexts, but not yet specifically demonstrated for HER2-positive drug resistance [58,59].

While early reports support the potential of exosomal lncRNAs such as LINC00969 and HOTAIR as liquid-biopsy candidates in HER2-positive breast cancer, broader clinical translation will require prospective validation in well-annotated cohorts with clinically meaningful endpoints, confirmation across representative patient subgroups, including HR-defined disease and different treatment contexts, and demonstration of incremental value beyond established clinicopathologic factors and existing liquid-biopsy markers. In parallel, analytical validity must be established using locked pre-analytical workflows, standardized EV isolation/lncRNA quantification procedures, and inter-laboratory reproducibility testing before these assays can be considered for clinical decision-making.

From an assay-development perspective, variability in EV separation and downstream RNA workflows can influence yield/purity and contribute to inconsistent EV-associated lncRNA quantification across studies, motivating harmonized and transparently reported protocols [60,61,62,63]. Community standards such as MISEV and transparency frameworks such as EV-TRACK provide practical guidance for fit-for-purpose EV characterization and method reporting to improve cross-study comparability and reproducibility. Accordingly, clinical-grade implementation will benefit from locked SOPs spanning pre-analytics, EV separation, and lncRNA quantification, ideally supported by inter-laboratory reproducibility testing.

### 4.2 Therapeutic Targeting Strategies

Given their regulatory position at critical nodes of drug resistance networks, certain lncRNAs might be directly targeted to help restore sensitivity to HER2-targeted therapies. Several approaches include antisense oligonucleotides (ASOs) and small interfering RNAs (siRNAs), nucleic acid-based molecules that selectively bind and degrade target lncRNAs [64], which could potentially suppress oncogenic transcripts such as HOTAIR or LINC00969, thereby mitigating their downstream effects on HER2 signaling and EMT [31,33]. Another emerging strategy involves small-molecule inhibitors that disrupt lncRNA–protein interactions, such as interfering with HOTAIR binding to PRC2 or LINC00969 interaction with HuR, which might offer greater specificity by sparing unrelated RNA functions. Additionally, exosome-mediated delivery systems could be leveraged to introduce tumor-suppressive lncRNAs into resistant tumors, exploiting the same intercellular communication channels that may currently facilitate resistance signals [65]. Preclinical validation of these strategies in HER2-positive models will be crucial, particularly in combination with existing HER2-targeted agents, to assess potential synergistic effects [66].

Together, these avenues illustrate how mechanistic insights into lncRNA-mediated resistance could be translated into clinical applications, although substantial preclinical and clinical validation will be required before such strategies become part of routine HER2-positive breast cancer management. Furthermore, safety and tissue specificity remain key translational constraints for lncRNA-targeting approaches. Because many lncRNAs act as network regulators, target knockdown may carry on-target toxicity in normal tissues, while ASOs can exhibit sequence-dependent off-target hybridization and chemistry-associated effects, and some CRISPR-Cas13 effectors have shown off-target RNA cleavage in eukaryotic systems [67,68,69]. Accordingly, clinical development will require careful candidate prioritization, tumor-selective delivery strategies, and transcriptome-wide off-target profiling during preclinical optimization. In addition, although lncRNA targeting may be synergistic with HER2-directed agents, dedicated preclinical studies are needed to define dosing/scheduling and to exclude additive or synergistic toxicity when combined with established anti-HER2 regimens.

Representative lncRNAs with demonstrated translational relevance in HER2-positive breast cancer are summarized in Table 2.

**Table 2:** Translational relevance of lncRNAs in HER2-positive breast cancer drug resistance.

Translational Aspect	LncRNA	Mode of Action	Clinical/Preclinical Evidence	Potential Application	References
**Circulating Biomarker**	HOTAIR (exosomal)	Elevated in plasma; correlates with HER2 positivity and poor clinical outcome	Detected in HER2+ patient plasma; associated with survival and chemotherapy response	Prognostic biomarker; monitoring of therapy resistance	[36]
**Circulating Biomarker**	LINC00969 (exosomal)	Binds HuR (ELAVL1) → stabilizes ERBB2/HER2 mRNA; elevated in plasma	Functional validation in cell models + patient plasma correlation with trastuzumab resistance	Predictive biomarker; liquid biopsy candidate	[33]
**Therapeutic Target**	HOTAIR	Recruits PRC2/EZH2 → silences PTEN; promotes EMT	Resistant HER2+ models; supported by clinical correlation	ASO/siRNA silencing; small-molecule disruption of HOTAIR–PRC2 interaction	[31,33]
**Therapeutic Target**	LINC00969	HuR-mediated stabilization of HER2 mRNA; exosome-mediated effects	Preclinical HER2+ resistance models; clinical association via plasma levels	ASO/siRNA silencing	[64,65,66]

Abb: long non-coding RNA (lncRNA); extracellular vesicles (EVs); antisense oligonucleotide (ASO); small interfering RNA (siRNA).

### 4.3 Prioritization Framework for Clinical Development

While multiple lncRNAs have been implicated in HER2-positive breast cancer resistance, translating these findings into clinical applications requires a systematic approach to prioritization. Given limited resources and the early stage of lncRNA-targeted therapeutics, we propose that candidate lncRNAs be evaluated against several key criteria to guide research and development efforts. First, strength of evidence in HER2-positive breast cancer: candidates with direct functional validation in HER2-positive models and clinical correlation in HER2-positive patient samples should be prioritized over those inferred from other cancer contexts. Second, clinical detectability and analytical validity: lncRNAs reliably detectable in accessible biofluids (plasma exosomes, serum) with established correlation to therapy resistance or clinical outcomes offer near-term biomarker feasibility. Third, functional non-redundancy: lncRNAs that regulate distinct, non-overlapping resistance mechanisms (e.g., HER2 mRNA stability vs. epigenetic silencing vs. immune evasion) provide unique therapeutic value and reduce the risk of compensatory bypass. Fourth, therapeutic tractability: candidates amenable to established modalities (ASO, siRNA, or CRISPR-Cas13 silencing), with favorable RNA structure, subcellular localization, and tissue accessibility, are more feasible for near-term therapeutic development. Fifth, dual utility as biomarker and therapeutic target: lncRNAs that can serve both diagnostic/monitoring roles and as intervention targets maximize translational impact and enable biomarker-driven stratification for targeted therapies. Finally, assay standardization feasibility: candidates for which robust, reproducible, and scalable measurement platforms can be developed (e.g., qRT-PCR, ddPCR, RNA-seq panels) are better positioned for clinical validation. Applying these criteria to the lncRNAs discussed in this review suggests a landscape of translational readiness (Table 3).

**Table 3:** Prioritization framework applied to candidate lncRNAs in HER2-positive breast cancer.

LncRNA	HER2+ Evidence Strength	Clinical Detectability	Functional Non-Redundancy	Therapeutic Tractability	Dual Biomarker/Target Utility	Assay Feasibility	Overall Priority Tier
**HOTAIR**	High	High	High	Medium	High	High	Tier 1 (Lead candidate)
**LINC00969**	High	High	High	Medium-High	High	High	Tier 1 (Lead candidate)
**GAS5**	Medium-High	Medium	Medium-High	Medium	Medium	Medium-High	Tier 2 (Promising)
**LncRNA-ATB**	Medium	Low	Medium	Medium	Medium	Medium	Tier 3 (Exploratory)
**SNHG1**	Low-Medium	Low	Medium	Medium	Low	Medium	Tier 3 (Exploratory)
**LINC00514**	Low-Medium	Low	Medium	Medium	Low	Medium	Tier 3 (Exploratory)

Note: Candidate lncRNAs are evaluated against six prioritization criteria relevant to clinical translation in HER2-positive breast cancer. Ratings (High/Medium/Low) reflect current evidence as summarized in Table 1 (mechanistic evidence) and Table 2 (translational relevance); HER2+: HER2-positive breast cancer; qRT-PCR: quantitative reverse-transcription PCR.

## 5 Discussion, Translational Perspective, and Future Direction

### 5.1 Potential LncRNA Mechanism Regulating Drug Resistance in HER2-Positive Breast Cancer

Drug resistance in HER2-positive breast cancer is frequently driven by dysregulation of key oncogenic pathways, including PI3K/Akt, MAPK/ERK, EMT, and immune evasion mechanisms. These pathways serve as central hubs for therapeutic response and are commonly altered in resistant tumors [70]. LncRNAs have been shown to modulate these same pathways in other cancer types through mechanisms such as ceRNA activity [71], transcriptional regulation [72], translation control [73], and cytokine signaling [74]. Although direct evidence in HER2-positive breast cancer remains limited, the ability of lncRNAs to modulate resistance-associated pathways across diverse cancers suggests that similar mechanisms may operate in this subtype. On this basis, we hypothesize that lncRNAs may contribute to targeted drug resistance in HER2-positive breast cancer by targeting these pathways through analogous mechanisms. The following section explores mechanistic insights from other cancer systems to support this hypothesis.

In erythroid differentiation, lncRNAs such as PCED1B-AS1 and DANCR coordinate with TFs like GATA1 and RUNX1 to dynamically regulate chromatin accessibility and gene expression [74,75]. While these TFs are hematopoietic-specific, uncovering such an lncRNA-TF-chromatin interaction network in HER2-positive breast cancer could reveal novel epigenetic mechanisms driving resistance and tumor progression. Additionally, evidence from other cancer types suggests that lncRNAs play critical roles in modulating drug resistance through the regulation of key signaling pathways. For instance, HOTAIR has been shown to promote chemoresistance via epigenetic silencing of tumor suppressor genes and activation of EMT-related transcriptional programs [41,76]. Similarly, MALAT1 enhances resistance to EGFR inhibitors in lung cancer by regulating EMT and the PI3K/AKT pathway [77]. UCA1, another well-characterized lncRNA, contributes to drug resistance by activating the AKT signaling cascade and promoting cell survival. These important pathways, such as EMT, PI3K/AKT, and epigenetic remodeling, are also implicated in resistance to HER2-targeted therapies such as trastuzumab and lapatinib. Therefore, it is possible that lncRNAs like HOTAIR, MALAT1, and UCA1 may similarly influence therapeutic response in HER2-positive breast cancer by modulating these conserved resistance mechanisms [31,78]. Further investigation into their expression patterns and functional roles in this context could uncover novel biomarkers or therapeutic targets to overcome drug resistance.

Another potential mechanism of lncRNAs regulating drug resistance in HER2 breast cancer could be further explored under ceRNA networks and the miRNA sponging mechanism. In HER2 positive gastric cancer, LINC00665 promotes trastuzumab resistance by sponging miR-199b-5p, leading to SERPINE1 upregulation and activation of the PI3K/Akt pathway [79]. Although this axis has not yet been validated in breast cancer, it underscores the potential for analogous lncRNA-miRNA-mRNA circuits to drive resistance across HER2-amplified tumors. Similarly, HOTAIR has been shown to sponge miR-331-3p, a microRNA that directly suppresses ERBB2 mRNA [80]. While this interaction remains hypothetical in breast cancer, it represents a plausible regulatory mechanism worthy of further investigation.

Beyond transcriptional and post-transcriptional regulation, some lncRNAs modulate mRNA translation by interacting with ribosomal components or translation initiation factors. Certain lncRNAs inhibit translation by sequestering initiation factors or disrupting ribosome assembly, while others enhance translation by stabilizing initiation complexes or facilitating ribosome recruitment [73,81]. These mechanisms allow cancer cells to selectively prioritize the synthesis of oncogenic proteins under stress conditions, contributing to adaptive resistance. Although such translation regulatory roles have not yet been confirmed in HER2-positive breast cancer, they could potentially affect HER2 protein levels or the expression of downstream effectors, thereby influencing therapeutic response.

LncRNAs also play a pivotal role in shaping the TME [82,83]. In various cancers, they regulate cytokine and chemokine signaling to promote immune evasion, stemness, and metastatic potential. For example, certain lncRNAs have been shown to modulate STAT3 signaling, either by enhancing its activation or stabilizing its downstream targets, thereby promoting EMT and suppressing immune surveillance [83,84]. Although the IL-6/JAK2/STAT3 axis has been reported to drive trastuzumab resistance in HER2-positive breast cancer, particularly in hormone receptor-negative subtypes, by promoting autocrine signaling and downstream survival pathways [85], direct links between lncRNAs and IL-6 regulation in this context remain underexplored. Nonetheless, it is possible that lncRNA-mediated cytokine signaling may contribute to both resistance and immune escape, suggesting further investigation into their role as potential therapeutic targets.

### 5.2 Translational Perspective

Resistance-associated lncRNAs such as LINC00969 and HOTAIR are overexpressed in HER2-positive breast cancer and have been linked to poor prognosis and trastuzumab resistance [33,86]. These molecules can be detected in biofluids, suggesting their potential use as minimally invasive biomarkers. In addition to lncRNA-based liquid biopsy, ctDNA has emerged as a complementary biomarker for predicting response to HER2-targeted therapies [87,88]. Combining ctDNA mutation profiling with lncRNA expression analysis may enhance early detection of resistance and guide adaptive treatment strategies. We hypothesize that integrating lncRNA expression analysis into liquid biopsy platforms could enable real-time, non-invasive monitoring of therapeutic response and facilitate early detection of resistance in HER2-positive breast cancer.

In the broader landscape of gene editing, CRISPR-Cas9 has emerged as a powerful discovery tool for uncovering drug resistance mechanisms in breast cancer. Genome-wide Cas9 knockout screens have successfully identified key coding genes that drive resistance to HER2-targeted therapies, such as trastuzumab and T-DM1, by systematically disrupting gene function at the DNA level [89,90,91]. However, the therapeutic application of Cas9 for targeting non-coding RNAs presents specific challenges; because Cas9 induces permanent genomic DNA cleavage, it carries a risk of off-target mutations and irreversible genomic instability. Furthermore, disrupting lncRNA loci at the genomic level does not always guarantee loss of transcript function. To address these limitations, recent attention has shifted toward RNA-targeting CRISPR systems for therapeutic purposes.

Unlike CRIPR-Cas9, CRISPR–Cas13 is an RNA-guided endonuclease that can specifically degrade target RNA transcripts, offering a precise and reversible means to silence oncogenic lncRNAs post-transcriptionally [92,93,94]. Although not yet applied in HER2-positive breast cancer, Cas13-based knockdown has been effective at silencing lncRNAs in tumor models and altering malignant phenotypes [95,96]. Moreover, multiple lncRNAs are established modulators of anticancer drug response [97]. We hypothesize that Cas13-mediated silencing of resistance-associated lncRNAs, such as HOTAIR or LINC00969, could disrupt survival pathways and enhance the efficacy of HER2-targeted therapies.

Recent findings by Barok et al. (2018) demonstrated that HER2-positive exosomes can bind and transfer trastuzumab–emtansine (T-DM1) to other HER2-positive cancer cells, retaining cytotoxic activity and highlighting the potential of exosomes as targeted delivery vehicles [98]. Building on this concept, HER2-targeted exosomes could be engineered to co-deliver HER2-directed therapeutics together with lncRNA-silencing cargo, offering a novel strategy to enhance treatment precision and overcome resistance [99,100,101]. Among potential targets, the lncRNA HOTAIR stands out due to its established role in association with EMT programs and therapy resistance phenotypes [41,42]. Knockdown of HOTAIR has been shown to sensitize HER2-positive cells to trastuzumab, and in gastric cancer, it has been reported to regulate HER2 expression via miR-331-3p sponging, suggesting a possible, though unconfirmed, regulatory axis in breast cancer as well [80]. Given that primary non-response and acquired resistance to trastuzumab are common, and that a substantial proportion of patients fail to achieve durable benefit on initial therapy [102,103], a dual-delivery approach combining trastuzumab (or T-DM1) with HOTAIR-silencing oligonucleotides in the same HER2-targeted exosome could offer a proactive means of suppressing resistance-associated pathways from the outset. This strategy holds translational promise as a modular platform for combinatorial targeting, with potential to improve therapeutic efficacy and delay resistance in HER2-positive breast cancer.

Beyond direct targeting of lncRNAs, emerging evidence highlights the role of the immune system in modulating response to HER2-targeted therapies [104]. Compared to hormone receptor–positive/HER2-negative tumors, HER2-positive breast cancers often exhibit higher immunogenicity, with increased tumor-infiltrating lymphocytes (TILs) and elevated programmed death-ligand 1 (PD-L1) expression observed in a substantial subset of patients [105,106,107,108]. Trastuzumab itself exerts part of its effect through ADCC, and immune suppression within the TME may blunt this response [109,110]. Resistance-associated lncRNAs such as HOTAIR have also been implicated in immune evasion, suggesting that lncRNA silencing could restore immune sensitivity [31,111]. Combining lncRNA-targeting approaches with immune checkpoint inhibitors or anti-HER2 vaccines may therefore offer a synergistic strategy to enhance antitumor immunity and overcome therapeutic resistance.

In addition, lncRNAs can contribute to immune evasion by suppressing antigen-presentation pathways. While neoantigens arise from tumor-specific mutations and are presented on MHC-I molecules, effective T-cell recognition requires an intact antigen-processing machinery (e.g., TAP1/TAP2, tapasin, β2-microglobulin) [112,113,114]. In breast cancer, the lncRNA LINK-A has been shown to downregulate MHC-I antigen presentation by promoting degradation of peptide-loading complex components, thereby reducing tumor visibility to CD8^+^ T cells [115]. HOTAIR has been reported to recruit PRC2 in cancer models [32], and has also been implicated in immune escape [116,117] (including PD-L1 induction via NF-κB), suggesting that HOTAIR or related lncRNAs could also repress antigen-presentation programs. Silencing such lncRNAs may restore antigen presentation and enhance immunogenicity, potentially improving responses to checkpoint inhibitors or HER2-targeted vaccines.

### 5.3 Future Direction

Current liquid-biopsy monitoring in HER2-positive breast cancer focuses primarily on ctDNA to track ERBB2 amplification, co-driver mutations, and molecular response. We propose augmenting this with exosomal lncRNAs measured from the same plasma draw, creating an orthogonal, dual-modality assay. Whereas ctDNA captures genomic change, exosomal lncRNAs report regulatory/phenotypic state (e.g., EMT, immune evasion, HER2 stabilization), potentially providing earlier and more mechanistically informative signals of emerging resistance. This proposed complementarity is summarized in Table 4.

**Table 4:** Proposed complementarity of ctDNA and EV/lncRNAs for early resistance detection in HER2-positive breast cancer.

Modality	Primary Signal Captured	What It May Add for Resistance Monitoring
ctDNA	Tumor-derived genomic alterations (e.g., copy-number changes, mutations)	Supports tracking clonal evolution and genomic drivers of resistance under therapy
EV/lncRNAs (exosomal lncRNAs)	Vesicle-associated regulatory RNAs reflecting transcriptional/post-transcriptional state	May report non-genomic resistance programs not necessarily coupled to new DNA alterations (e.g., EV LINC00969 linked to HER2 mRNA stabilization via HuR and trastuzumab resistance)

Abb: circulating tumor DNA (ctDNA).

Prospective studies are warranted to evaluate whether integrated ctDNA + EV/lncRNA models improve early detection of resistance compared with either modality alone. Such studies could pre-specify pCR (neoadjuvant) and early molecular progression/PFS (metastatic) as endpoints and quantify incremental performance using multivariable models.

We propose pre-specifying pathological complete response (pCR) in the neoadjuvant setting and early molecular progression (EMP)/progression-free survival (PFS) in the metastatic setting as primary outcomes, and to test whether a combined ctDNA + exosomal-lncRNA panel improves predictive performance over either modality alone. Standardization of pre-analytics (collection tubes, processing times, isolation methods) and harmonized analytic pipelines will be essential to ensure cross-site reproducibility and enable clinical adoption. Clinical translation will also require analytically validated assays for exosomal lncRNAs and ctDNA with locked standard operating procedures (SOPs). Stratification frameworks may consider hormone-receptor status, PIK3CA mutation, baseline TILs/PD-L1, and early on-treatment molecular changes to guide escalation toward lncRNA-targeting combinations when resistance signals emerge. For combination strategies with HER2-directed agents, dose- and schedule-finding studies should explicitly track overlapping toxicities (e.g., hepatic/gastrointestinal (GI) toxicity with tyrosine kinase inhibitors (TKIs), hematologic toxicity with antibody-drug conjugates (ADCs), and cardiac monitoring with trastuzumab-based regimens) alongside pharmacodynamic knockdown and antitumor efficacy. Given the recognized risk of cardiac dysfunction with trastuzumab and related HER2-directed agents [118,119], we additionally recommend embedding exploratory cardio-oncology readouts within the same plasma workflow, specifically, circulating exosomal lncRNAs indicative of cardiac stress, to enable early detection and mitigation without additional sampling.

Future studies should determine whether resistance-associated lncRNAs reshape antitumor immunity by modulating antigen processing and presentation, checkpoint ligand induction, and interactions with effector cells (NK, macrophages, T cells). Causal loss-of-function studies (CRISPR interference (CRISPRi), antisense oligonucleotides, Cas13) in HER2-positive models can be paired with orthogonal immune readouts and validated in humanized-immune xenografts in combination with anti-HER2 ± PD-1/PD-L1 blockade; single-cell or spatial profiling of patient tumors can then localize these effects *in situ*. Collectively, these approaches can help identify actionable lncRNAs, guide rational combination strategies, and define practical biomarkers for clinical testing.

Therapeutic delivery remains a translational bottleneck even with HER2-targeted exosomes. Receptor binding alone does not ensure efficient tumor deposition, endosomal escape of RNA cargo, or batch-to-batch reproducibility [120,121,122]. Future work should standardize quantitative readouts for biodistribution and tumor selectivity, on-target knockdown in tumor tissue, cytosolic delivery and release kinetics, and innate immune activation/opsonization, alongside manufacturing quality-control (QC). Building on the observation that HER2-positive exosomes can transfer T-DM1 with retained cytotoxicity, engineered exosomes co-loading lncRNA silencers with anti-HER2 agents should be tested against simple co-administration to determine whether single-particle delivery improves intratumoral co-exposure without adverse effects on pharmacology. Because nucleic-acid therapeutics and RNA-targeting nucleases can trigger off-target and innate-immune effects, these delivery studies should include cytokine-release/innate-immunity panels, transcriptome-wide off-target assessment, and good laboratory practice (GLP)-style toxicology [67,123].

Given the immune contribution to anti-HER2 efficacy, lncRNA silencing should be evaluated in combination with checkpoint inhibitors and HER2-directed therapies in humanized-immune models. To define optimal sequencing, studies should compare lncRNA “priming” (silencing before anti-HER2 ± immune checkpoint inhibitor (ICI)) versus concurrent administration, with a focused biomarker set capturing antigen presentation, checkpoint dynamics, and effector function. Parallel monitoring of escape mechanisms like loss of HER2 and durable antigen-processing machinery defects will inform rational sequencing and companion diagnostics.

Finally, a curated, open resource linking lncRNAs to targets, immune phenotypes, and therapeutic responses in HER2-positive disease would accelerate discovery. Harmonized metadata, sample handling (collection tubes, time-to-spin, storage, freeze–thaw cycles), EV isolation (method, yield, CD9/CD63/CD81 markers, particle counts), RNA workflows (extraction kit, spike-in controls, library preparation, unique molecular identifiers (UMIs) usage), and analytic settings (normalization, batch correction, reference versions), should accompany all datasets alongside key clinical covariates (hormone-receptor status, prior therapies, TILs, PD-L1 assay/score, ERBB2 copy number/mutation, response endpoints). Adopting FAIR (Findable, Accessible, Interoperable, Reusable) data principles and reporting checklists will support reproducibility [124,125].

## 6 Conclusion

Long non-coding RNAs are emerging as key regulators of therapeutic response in HER2-positive breast cancer, with evidence implicating resistance-associated lncRNAs such as LINC00969 and HOTAIR. This review synthesizes current evidence on established mechanisms and advances potential hypotheses for underexplored mechanisms of lncRNA-mediated resistance. We also outline potential translational pathways, including dual-modality liquid biopsy approaches that integrate exosomal lncRNAs with ctDNA, as well as HER2-targeted exosomal co-delivery of lncRNA silencers with anti-HER2 agents. Furthermore, we propose focused future directions involving causal mechanistic studies, delivery optimization with standardized readouts, and prospective clinical validation with pre-specified endpoints.

Together, this review consolidates existing knowledge in the field and provides a framework for advancing the translational application of lncRNAs in HER2-positive breast cancer management.

## Data Availability

Not applicable.
